# South Indian Children’s Neurodevelopmental Outcomes After Group B Streptococcus Invasive Disease: A Matched-Cohort Study

**DOI:** 10.1093/cid/ciab792

**Published:** 2021-11-03

**Authors:** Hima B John, Asha Arumugam, Mohana Priya, Nandhini Murugesan, Nandhini Rajendraprasad, Grace Rebekah, Proma Paul, Jaya Chandna, Joy E Lawn, Sridhar Santhanam

**Affiliations:** 1 Department of Neonatology, Christian Medical College, Vellore, India; 2 Department of Biostatistics, Christian Medical College, Vellore, India; 3 Maternal, Adolescent, Reproductive & Child Health (MARCH) Centre, London School of Hygiene and Tropical Medicine, London, United Kingdom; 4 Department of Infectious Disease Epidemiology, London School of Hygiene and Tropical Medicine, London, United Kingdom

**Keywords:** neurodevelopmental impairment, group B Streptococcus invasive disease, India, neurodevelopmental outcomes

## Abstract

**Background:**

This study is part of a multicountry matched-cohort study designed to estimate the risk of long-term neurodevelopmental impairment (NDI) of children exposed to invasive group B Streptococcus (iGBS). The specific objective of this paper is to compare NDI across domains of iGBS survivors with a matched non iGBS group in our population.

**Methods:**

Survivors of iGBS in a South Indian hospital were identified and recruited between January 2020 and April 2021. Cases were compared with age- and gender-matched non iGBS children. Participants were assessed using Bayley Scales of Infant and Toddler Development–3rd edition (BSID-III), Wechsler Preschool and Primary Scale of Intelligence–4th edition (WPPSI-IV), Wechsler Intelligence Scale for Children–5th edition (WISC-V), Child Behavior Checklist (CBCL), and Bruininks-Oseretsky Test of Motor Proficiency, 2nd edition (BOT-2), depending on age.

**Results:**

Our cohort comprised 35 GBS-exposed and 65 matched non iGBS children, aged 1–14 years. The iGBS-exposed group had 17 (48.6%) children with impairment in ≥1 domain compared to 25 (38%) in the non iGBS group (unadjusted OR, 1.51; 95% CI, .65–3.46), 9 (26%) children with “multi-domain impairment” compared to 10 (15.4%) in the non iGBS group (unadjusted OR, 1.90; 95% CI, .69–5.24), and 1 (2.9%) child with moderate to severe impairment compared to 3 (4.6%) in the non iGBS group (unadjusted OR, .60; 95% CI, .06–6.07). In the iGBS group, more children had motor impairments compared with the non iGBS group (unadjusted OR, 10.7; 95% CI, 1.19–95.69; *P* = .034).

**Conclusions:**

Children with iGBS seem at higher risk of developing motor impairments compared with a non iGBS group.

**KEY FINDINGS**

**1. What Is Known and What Is New?**

There are an estimated 392 000 children worldwide with iGBS, with the highest numbers in sub-Saharan Africa and Asia. Globally, there are almost no published studies of NDI among iGBS survivors from LMICs. This is the first study from Asia to examine neurodevelopment of iGBS survivors using standardized developmental assessment tools across several domains.

**2. What Did We Do and What Did We Find?**

We identified 35 survivors of iGBS aged 1–14 years and 65 matched non iGBS children, undertaking standardized assessments of NDI. The iGBS-exposed children had a trend towards greater NDI, but this was not statistically significant (unadjusted OR: 1.51; 95% CI: .65–3.46). An important limitation of this study was that some children were not able to come due to travel restrictions during the COVID-19 pandemic, reducing the capture especially of both iGBS and non iGBS cohort.

**3. What To Do Now in PROGRAMMES?**

Early interventional services and follow-up programmes are required for survivors of iGBS to optimize neurodevelopmental outcomes.

**4. What’s Next in Research?**

There is a need for culturally appropriate measurement tools, especially in measurement of language and cognition. Larger studies are needed to estimate the incidence of NDI in this population and to study environmental contributors to impairment.

The estimated incidence of severe neonatal infections in low- and middle-income countries (LMICs) is 6.9 million [1]. Invasive group B Streptococcus disease (iGBS), an infection that presents as sepsis or meningitis, is a leading cause of neonatal sepsis with a reported incidence of 0.49 per 1000 live births [2, 3].

Intrauterine and neonatal insults contribute to a high risk of developing long-term neurodevelopmental impairment (NDI) including cognitive, motor (eg, cerebral palsy), hearing, and visual impairment domains [4]. Bacterial meningitis, especially in neonates, is a notable cause of NDI, with pathophysiological disruptions such as cerebral inflammation and edema [5]. After neonatal meningitis, an estimate of moderate to severe NDI is 23% (95% confidence interval [CI]: 19–26%) [6]. A systematic review of NDI of group B Streptococcus (GBS) survivors found moderate to severe NDI in 18% of meningitis survivors, but no usable data after iGBS sepsis. This review included 18 studies from upper- and middle-income countries with a paucity of data for patients older than 2 years, and recommended future studies assessed the total burden of GBS disease in older children and assessing all developmental domains using valid assessment tools [7]. There are no published studies that assessed neurodevelopment outcomes of iGBS survivors in an Asian population.

## AIM AND OBJECTIVES

This article is part of a series ‘Every Country, Every Family: Group B Streptococcal Disease Worldwide’ reporting a value proposition for maternal vaccines against GBS by the World Health Organization (WHO). The aim of this paper is to present data collected in India as part of a multicountry study describing neurodevelopmental outcomes of iGBS survivors by domains of cognition, language, motor skills, and behavior and comparison with a non iGBS group. 

The objectives of the paper are as follows: (1) to describe a cohort of iGBS survivors and a matched non iGBS cohort and (2) to evaluate the risk of NDI and categorize severity in the domains of vision, hearing, cognition, language, motor skills, and behavior in the iGBS cohort when compared with a non iGBS group.

## METHODS

### Setting

**Figure 1.**
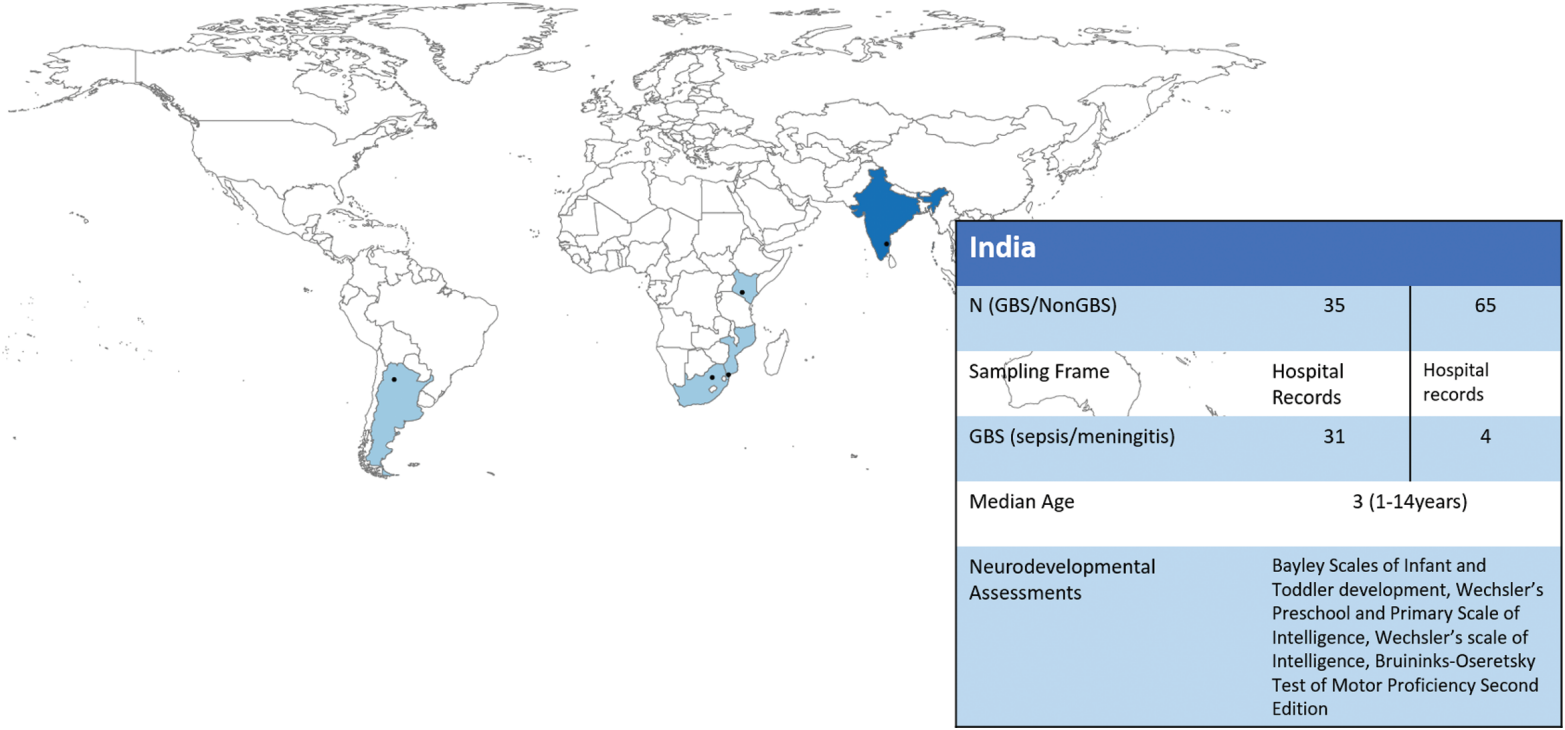
Map of the multicountry iGBS long-term follow-up studies, showing details of the India site. India was 1 of 5 low- and middle-income countries who participated in the study. Abbreviations: GBS, group B Streptococcus; iGBS, invasive group B Streptococcus.

The study setting was a tertiary care teaching hospital in South India (Figure 1). This hospital is a referral perinatal center catering to the population of 4 adjoining districts of 3 neighboring states. The 75 bedded neonatal unit with level 3 facilities has admissions of over 2500 infants every year. There are approximately 12 000–14 000 births per year and the neonatal mortality rate between 2015 and 2019 ranged from 2.7 to 5.6 per 1000 live births (mean: 3.8/1000 live births; national Neonatal Mortality Rate (NMR) in 2019: 21.7/1000 live births). The hospital has a risk-based intrapartum antibiotic policy for GBS prophylaxis since 2003, with no national policy for the same. A multinational study in 2017 found the prevalence of GBS colonization among pregnant women in the institution was 20.9 % [8]. The incidence of GBS disease between 1998 to 2010 in the institution was 0.76 per 1000 live births, of which the incidence of early-onset sepsis was 0.68 per 1000 live births (95% CI: .52–.83) [8, 9].

### Study Design

The study was a matched-cohort study. Exposed were survivors of infant iGBS disease, henceforth termed as “iGBS survivors,” and unexposed were individuals with no GBS identified. This study is part of a multicenter trial on the long-term outcomes of GBS survivors in LMICs; the study protocol has already been published [10].

### Participants

Survivors of iGBS born between August 2006 and July 2018 were identified from the hospital’s database. As per protocol, children born at less than 32 weeks’ gestation were excluded. An additional criterion for inclusion was knowledge of English or Tamil for administration of neurodevelopmental assessments.

Parents were contacted by telephone and via post with an invitation to attend the neurodevelopmental assessment. Home visits, although initially planned, were not executed due to the coronavirus disease 2019 (COVID-19) pandemic during the period of recruitment. Exposure to GBS disease was defined as GBS isolated in blood culture between 0 and 89 days of the infant’s life. This study did not differentiate between GBS sepsis or GBS meningitis. In our cohort, none of the infants with “meningitis” had a positive cerebrospinal fluid (CSF) culture, which is the gold standard for diagnosis, but were diagnosed based on high CSF counts or protein.

Non iGBS children were matched for gender and age of ±2 months of the iGBS survivors. We aimed to recruit 3:1 non-GBS to exposed children. Non iGBS children were recruited by identifying and contacting case-matched children from the hospital records, via the immunization clinic and through distribution of posters in the community. No exclusion criteria were placed, except for a positive history of GBS infection in early infancy. There was a time lag in the recruitment of exposed children due to government-imposed lockdown measures to contain COVID-19 infection.

Recruitment was from January 2020 to April 2021. Once the family arrived at the hospital, written informed consent and child assent were obtained. The demographic, health, and economic health questionnaire and the EQ5D questionnaire were administered to the caregiver. The children’s anthropometric measurements were taken, vision and hearing assessed, and the neurodevelopmental assessments and relevant questionnaires administered. Information was collected on paper forms, then transferred using standard operating procedures to a tablet-based application for data entry.

The study assessment team consisted of 3 occupational therapists, 2 psychologists, a fieldworker, and a data entry operator. All team members were trained using an 8-session training module, covering standard guidelines for assessment of anthropometry, vision, and hearing, and administration of questionnaires and data entry. They were also certified in the administration, interpretation, and scoring of the assessment tools.

#### Neurodevelopmental Assessments

##### Children *A*ged *B*elow 42 *M*onths.

 Children aged 42 months or below were assessed using the Bayley Scales of Infant and Toddler Development, 3rd edition (BSID-III), for cognition, language, motor skills, socioemotional skills, and adaptive behavior. The BSID is a globally accepted goal standard in child assessment and has recently been used in a large number of studies in our population [11–13].

##### Children Aged Above 42 Months.

 In children older than 42 months, cognition was assessed using the Wechsler’s Preschool and Primary Scale of Intelligence–4th edition (WPPSI-IV) or the Wechsler’s Scale of Intelligence–5th edition (WISC-V), depending on age. The WPPSI assesses cognitive abilities in children aged 2.5 to 7 years. It has 13 subtests, which yield scaled scores, standard scores, and percentiles [14]. The WISC-V is a commonly used assessment in school-aged children. Both tools render composite scores for fluid reasoning, processing speed, verbal comprehension, visual spatial and working memory and a full-scale IQ. The validity of both scales has been supported for use in LMICs and the WISC-IV standardized to the Indian population [15, 16].

Motor skills were assessed using the Bruininks-Oseretsky Test of Motor Proficiency, 2nd edition (BOT-2). It assesses fine and gross motor skills in children and youth aged 4 to 21 years. This study used the short form of the assessment, which yields standard scores, percentile ranks, and descriptive categorization [17].

Behavioral outcomes for all children were measured using the Child Behavior Checklist (CBCL). The CBCL is a parent-report questionnaire (composed of 2 versions: a younger 1.5–5 years and older 6–18 years) on which the child is rated on various behavioral and emotional problems. It assesses internalizing (ie, anxious, depressive, and overcontrolled) and externalizing (ie, aggressive, hyperactive, noncompliant) behaviors [18, 19]. The questionnaire was translated and back-translated into Tamil. Questions were read aloud to caregivers with limited literacy.

The assessors were not blinded to participant group. During a developmental or psychological assessment, it is inevitable that parents share their traumatic neonatal intensive care unit experience and this is not discouraged since this information contributes to the holistic understanding of the child. Hence, the assessors were not blinded to the groups. Since the assessment tools have stringent guidelines on scoring and interpretation, we did not anticipate bias due to the lack of blinding. Parental concerns were addressed by the team. Children with mild NDI had an additional appointment with the unit’s psychologist or occupational therapist (depending on the domain of impairment). Parents were taught a home-based program and encouraged to attend regular follow-up visits at 2-month intervals. Children with moderate to severe NDI were referred to the institution’s Developmental Pediatric Unit.

All assessments were scored according to their scoring manuals. Neurodevelopmental impairment was defined based on the work by the Global Burden of Disease [7]. Severity coding within each assessment was as follows (Supplementary Table 2): Mild, if 1–2 SDs below the standardized mean; Moderate, if 2–3 SDs below the standardized mean; Severe, if 3 or more SDs below the standardized mean; “Any impairment,” if the child had impairment in any domain (vision, hearing, cognition, language, motor, or behavior); “Multi-domain impairment,” if there were impairments in more than 1 domain; “Moderate to severe impairment,” if the child had moderate or severe impairment in any domain [10]. Socioeconomic status was assessed using the Updated Modified Kuppuswamy SES scale for the year 2020 [20].

### Statistical Methods

Neurodevelopmental outcomes were assessed separately for the 2 age groups—1 group with children younger than 42 months (who had BSID and CBCL assessments) and 1 group with children 43 months and older (who had a WISC or WPPSI, BOT, and CBCL assessments)—and combined (Supplementary Table 2). Domains of vision, hearing, cognition, language, motor abilities and behavior were compared between exposed and non-GBS groups using a severity classification for NDI.

Analysis was undertaken in SPSS software version 21 (IBM Corporation). Descriptive statistics were reported using means ± SDs for continuous variables; categorical variables were reported using frequencies and percentages. Association was reported using chi-square/Fisher’s exact test. Comparison of means was reported using 2 independent-samples *t* tests. Binary logistic regression was performed to arrive at the risk factor analysis. The odds ratio (OR) was reported along with the 95% C). A *P* value less than .05 was considered statistically significant.

## RESULTS

### Objective 1: Description of GBS Survivors and a Matched Non-GBS Group

**Figure 2.**
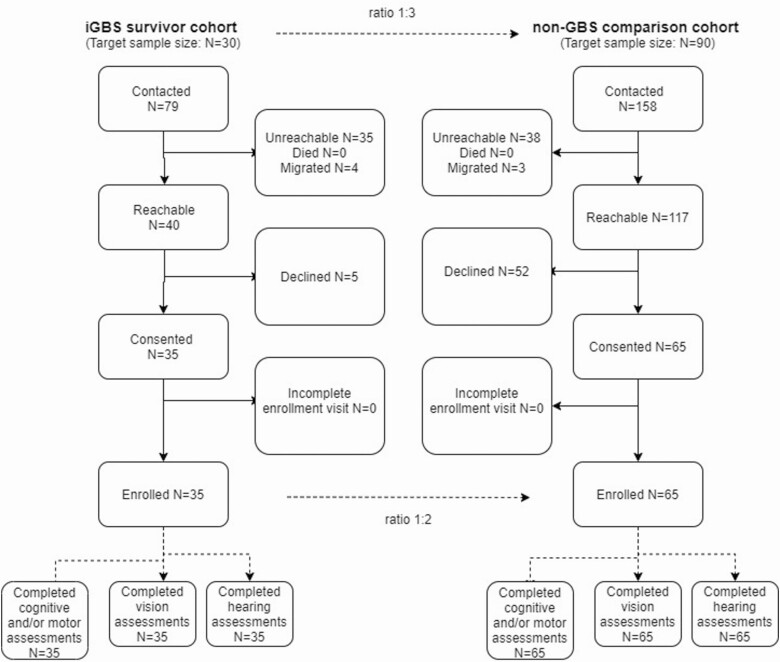
Participant flow of iGBS cases and non iGBS children recruited. Out of 79 iGBS survivors contacted, 35 consented for participation and completed the assessment. Out of 158 matched non iGBS children contacted for participation, 65 children consented and completed neurodevelopmental, vision and hearing assessments. Abbreviations: GBS, group B Streptococcus; iGBS, invasive group B Streptococcus.

Out of 79 iGBS survivors contacted, 35 (44.3%) consented for participation and completed the assessment (Figure 2). Of the 35 children with iGBS, 33 (94.3%) had early-onset sepsis and 2 (5.7%) had late-onset sepsis. There were no significant differences in gender, prematurity, birth weight, gestational age, onset of sepsis, and rates of meningitis and chorioamnionitis between children who consented for participation and those who did not participate. Out of 158 matched non iGBS children approached for participation, 65 (41.1%) children consented and completed the assessments (Figure 2). One child in the non iGBS group had sepsis not caused by GBS in the neonatal period. Initially, it was planned to recruit patients from the hospital database using sequential sampling based on closest match. We were able to recruit approximately one-third of the sample in this way. Due to the pandemic-related travel restrictions, we later used convenience sampling by offering assessments to children who came for an immunization visit and distribution of posters in the community.

Participants were aged 1–14 years (mean [SD]: 4.49 [3.47]; median: 3 years). There were no significant differences in demographic variables in exposed and non iGBS groups except in birth order (*P* = .003) (Table 1). Of the parents of children who underwent treatment for GBS sepsis in our hospital who were contacted, none reported postdischarge death.

**Table 1.** Demographic and Health Characteristics Among Survivors of Invasive Group B Streptococcus in Infancy and a Comparison Cohort in India

	iGBS Cohort (n = 35)	Non iGBS Cohort (n = 65)	P
Matching criteria			
Age at assessment, mean (SD), months	62.9 (46)	56.6 (38)	.46
Sex, n (%)			.67
Female	19 (54)	39 (60)	
Male	16 (45)	26 (40)	
iGBS characteristics			
Clinical syndrome, n (%)			
Sepsis	35 (100)		
Meningitis	4 (11)		
GBS onset, n (%)			
Early onset (0–6 days)	33 (94)		
Late onset (7–89 days)	2 (5)		
Birth history			
Birth weight, mean (SD), g	2916 (510)	2944 (570)	.81
Gestational age (in weeks), n (%)			
≥37	31 (88)	62 (95)	.23
33–36	4 (11)	3 (4)	
Birth order, n (%)			
First born	30 (85)	35 (53)	<.01
Second born or higher	5 (14)	30 (46)	
Caregiver and household characteristics			
Highest education for main caregiver, n (%)			
Illiterate/primary/middle school	6 (17)	12 (18)	.23
High school/intermediate	7 (20)	13 (20)	
Technical/graduate and above	22 (62)	40 (61)	
Carer employment status, n (%)			
Housework	28 (80)	41 (63)	.02
Income from paid employment	7 (20)	24 (36)	
Family structure, n (%)			
Joint family	11 (31)	28 (43)	.28
Nuclear family	24 (68)	37 (56)	
Residential classification, n (%)			.28
Urban	18(51)	42(64)	
Rural	17(48)	23(35)	
Head of household education, n (%)			
Illiterate/primary/middle school	10 (28)	15 (23)	.07
High school/intermediate	8 (22)	24 (36)	
Technical/graduate and above	17 (48)	26 (40)	
Head of household occupation, n (%)			
Unskilled	5 (14)	13 (20)	.59
Manual skilled	4 (11)	3 (4)	
Sales/clerical	14 (40)	21 (32)	
Professionals/ Managers	12 (34)	28 (43)	
Household monthly income, n (%)			
Less than Rs* 10 000	9 (25)	18 (27)	.054
Rs. 10 002–29 972	16 (45)	22 (33)	
Rs. 29 973–49 961	9 (25)	15 (23)	
More than Rs 49 962	1 (2)	10 (15)	
SES classification, n (%)			
Upper/upper middle	11 (31)	27 (41)	.29
Lower middle	14 (40)	17 (26)	
Upper lower/lower	10 (28)	21 (32)	

Abbreviations: GBS, group B Streptococcus; iGBS, invasive group B Streptococcus; SES, socioeconomic status; Rs, Rupees

### Objective 2: Risk of NDI in the GBS Cohort When Compared With the Non-GBS Group

**Figure 3.**
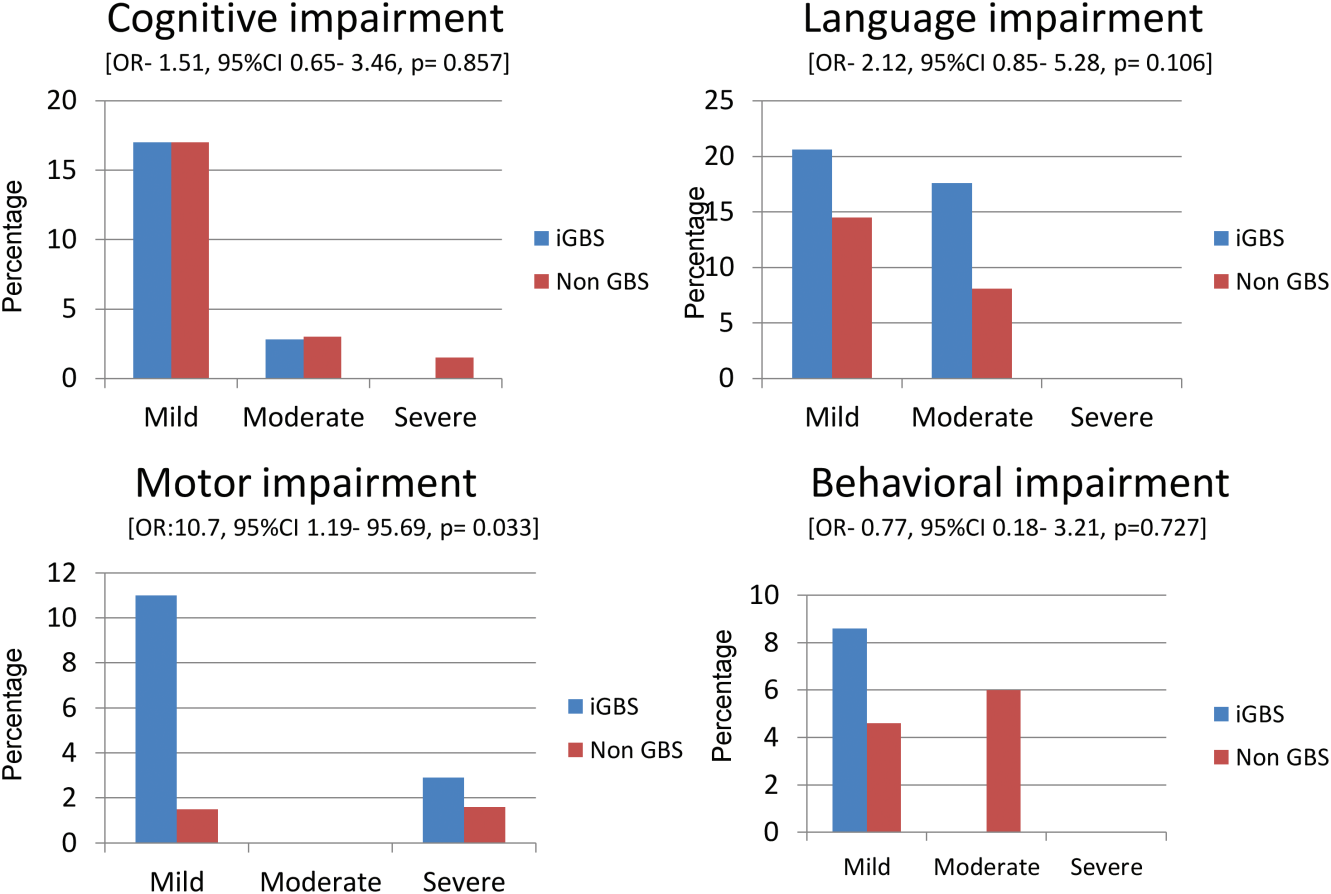
Results of NDI by domain for iGBS and non iGBS children. The figure describes impairment severity by domain in the iGBS and non iGBS group. Unadjusted OR values are reported for no impairment versus impairment. Abbreviations: CI, confidence interval; GBS, group B Streptococcus; iGBS, invasive group B Streptococcus; NDI, neurodevelopmental impairment; OR, odds ratio.

Of the 35 iGBS survivors who participated in the study, all children had GBS sepsis and 4 (11%) also had meningitis. Among iGBS survivors, 17 (48.6%) children had impairment in at least 1 of the assessed domains as compared to 25 (38%) in the non iGBS group (unadjusted OR: 1.51; 95% CI: .65–3.46); 9 (26%) children had impairment in more than 1 domain compared to 10 (15.4%) in the non iGBS group (unadjusted OR: 1.90; 95% CI: .69–5.24); and 1 (2.9%) child had moderate to severe impairment compared to 3 (4.6%) in the non iGBS group (unadjusted OR: .60; 95% CI: .06–6.07). The iGBS group had more children with motor impairments compared with the non iGBS group (unadjusted OR: 10.7; 95% CI: 1.19–95.69; *P* = .033). There were no differences in impairments in vision, hearing, cognitive skills (unadjusted OR: 1.51; 95% CI: .65–3.46; *P* = .857), language skills (unadjusted OR: 2.12; 95% CI: .85–5.28; *P* = .106), or behavior (unadjusted OR: .77; 95% CI: .18–3.21; *P* = .727) between the iGBS and non iGBS groups (Table 2, Figure 3).

**Table 2.** Developmental Outcomes Among Survivors of Invasive Group B Streptococcus Disease and a Comparison Cohort

	iGBS Cohort (n = 35)	Non-GBS Cohort (n = 65)	P
Children below 42 months, n	17	32	
Age at assessment, mean (SD), months	28.8 (8.3)	27.3 (8.9)	.636
Vision impairment	1 (6)	0	.347
Hearing impairment	1 (6)	0	.378
Bayley Scales of Infant and Toddler Development-3			
Cognition Composite score, mean (SD)	106.47 (17)	101.56 (10)	.215
Cognition mild impairment	0	2 (6)	.537
Language Composite score, mean (SD)	99.94 (13)	104.03 (11)	.504
Language moderate impairment	2 (11)	1 (3)	.124
Motor Composite score, mean (SD)	103.12 (7)	107.53 (10)	.266
Motor skills mild impairment	1 (6)	0	.347
Socio-emotional scale Composite score, mean (SD)	105 (27)	114 (17)	.170
Socio-emotional scale interpretation			
Mild impairment	3 (17)	2 (6)	.053
Severe impairment	2 (11)	2 (4)	
Adaptive behavior Composite score, mean (SD)	82 (20)	80 (10)	.654
Adaptive behavior scale interpretation			
Mild impairment	5 (29)	10 (31)	.459
Moderate impairment	2 (11)	9 (28)	
Severe impairment	4 (23)	7 (22)	
Child Behavior Checklist (n = 45)			
Internal interpretation			.504
Borderline	0	2 (7)	
Clinical range	1 (6)	1 (3)	
External interpretation			.664
Borderline	1 (6)	2 (7)	
Clinical range	1 (6)	4 (14)	
Total interpretation			
Borderline	1 (6)	2 (7)	.364
Clinical range	0	3 (10)	
Any impairment	6 (35)	9 (28)	.747
Any moderate/severe impairment	0	1 (3)	1.000
Multi-domain impairment	4 (23)	1 (3)	.043*
Children above 42 months, n	18	33	
Age at assessment, mean (SD), months	95.17 (43)	84.97 (34)	.145
Vision impairment	1 (6)	2 (6)	.718
Hearing impairment	0	0	
Wechsler’s Preschool and Primary Scale of Intelligence–4th edition (WPPSI- IV)/Wechsler’s Scale of Intelligence–5th edition (WISC-V)			
Fluid reasoning Composite score, mean (SD)	100.19 (12.6)	107.9 (15.1)	.553
Fluid reasoning interpretation (n = 45), n	16	29	.644
Mild impairment	1 (6)	1 (3)	
Moderate impairment	1 (6)	0	
Severe impairment	0	1 (3)	
Processing speed Composite score, mean (SD)	92.25 (13.1)	87.86 (14.0)	.401
Processing speed interpretation (n = 45)			
Mild impairment	4 (25)	9 (31)	.710
Moderate impairment	2 (12)	6 (20)	
Severe impairment	1 (6)	3 (10)	
Working memory Composite score, mean (SD)	100.76 (12)	107.57 (16)	.061
Working memory interpretation (n = 47)			
Mild impairment	2 (12)	3 (10)	.697
Moderate impairment	0	3 (10)	
Severe impairment	0	0	
Visual spatial Composite score, mean (SD)	95.12 (11.5)	95.20 (12.6)	.461
Visual spatial interpretation (n = 47)			
Mild impairment	3 (17)	7 (23)	.790
Moderate impairment	2 (11)	1 (3)	
Severe impairment	0	1 (3)	
Verbal comprehension Composite score, mean (SD)	89.18 (10.64)	91.93 (12.31)	.936
Verbal comprehension interpretation (n = 47)			
Mild impairment	5 (29)	8 (26)	.547
Moderate impairment	4 (23)	4 (13)	
Severe impairment	0	0	
Full-scale IQ Composite score, mean (SD)	93.78 (10.5)	99.39 (15.2)	.069
Full-scale IQ interpretation (n = 51)			
Mild impairment	3 (16)	9 (27)	.714
Moderate impairment	1 (5)	1 (3)	
Severe impairment	0	1 (3)	
Bruininks-Oseretsky Test of Motor Proficiency (BOT-2)			
Standard scores, mean (SD)	48.88 (10.9)	51.9 (8.6)	.380
Percentile rank, mean (SD)	47.47 (30)	54.74 (25)	.505
Interpretation (n = 48)			.047*
Mild impairment	3 (17)	1 (3)	
Moderate impairment	0	0	
Severe impairment	1 (6)	0	
Child Behavior Checklist			
Internal interpretation, n	18	33	.768
Borderline	1 (5)	4 (12)	
Clinical range	0	1 (3)	
External interpretation			
Borderline	1 (5)	1 (3)	1.0
Clinical range	1 (5)	1 (3)	
Total interpretation			
Borderline	2 (11)	1 (3)	.542
Clinical range	0	1 (3)	
Any impairment	11 (61)	16 (48)	.558
Any moderate/severe impairment	1 (5)	2 (6)	1.000
Multi-domain impairment	5 (27)	9 (27)	1.0
Age groups combined			
Vision impairment	1 (5)	2 (6)	1.0
Hearing impairment	1 (2)	0	.372
Motor skills (n = 97)			
Mild impairment	4 (11)	1 (1)	.033*
Moderate impairment	0	0	
Severe impairment	1 (2)	0	
Cognition			
Mild impairment	6 (17)	11 (17)	.908
Moderate impairment	1 (2)	2 (3)	
Severe impairment	0	1 (1)	
Language			
Mild impairment	7 (20)	9 (14)	.224
Moderate impairment	6 (17)	5 (8)	
Severe impairment	0	0	
Behavioral impairment			
Mild impairment	3 (8)	3 (4)	.252
Moderate impairment	0	4 (6)	
Any impairment	17 (48.6)	25 (38.5)	.397
Moderate to severe impairment	1 (2.9)	3 (4.6)	1.000
Multi-domain impairment	9 (25.97)	10 (15.4)	.285

Data are presented as n (%) unless otherwise indicated. Abbreviations: GBS, group B Streptococcus; iGBS, invasive group B Streptococcus. *Highlights statistical significance.

Looking at iGBS and non iGBS groups combined, among children with “any impairment,” 27 (64%) had language impairment, 20 (47%) had cognitive impairment, 6 (14%) had motor impairment, and 11 (26%) had behavioral impairment. Among children with “multi-domain impairment,” 19 (100%) had language impairment, 17 (89%) had cognitive impairment, 6 (31.5%) had motor impairment, and 3 (15.7%) had behavioral impairment. Among children with “moderate to severe impairment,” 3 (75%) had language impairment, 4 (100%) had cognitive impairment, and 1 (25%) had motor impairment.

#### Children Below 42 Months

There were 17 children in the exposed group and 32 in the non iGBS group below 42 months. Four children (23.5%) in the exposed group and 1 (3.1%) child in the non iGBS group had “multi-domain impairment’ (*P* = .043).

#### Children Above 42 Months

There were 18 children in the exposed group and 33 children in the non iGBS group above 42 months. The exposed group had more children with motor impairments: 4 (23.5%) in the exposed group and 1 (3.1%) child in the non iGBS group (*P* = .042). There was a trend towards lower scores in the working memory subscale (*P* = .06) and the full-scale IQ (*P* = .06) in the exposed group (Table 2) compared with the non iGBS group. When children with “any impairment” were excluded from analysis, the exposed group had lower full-scale IQ scores than the non iGBS group (*P* = .014).

## Discussion

This study found that iGBS-exposed children had approximately 50% higher odds of NDI but this was not statistically significant (unadjusted OR: 1.51; 95% CI: .65–3.46). Children in the iGBS group had more motor impairments compared with the non iGBS group (*P* = .033). An important limitation of this study was the small size due to COVID-19 pandemic travel restrictions, reducing the number of both iGBS and the non iGBS children recruited. This is the first published study in Asia examining NDI among iGBS survivors using standardized developmental assessment tools across several domains, and comparing with a matched non iGBS group.

The iGBS-exposed group had 17 (48.6%) children with “any impairment” compared to 25 (38%) in the non iGBS group, 9 (26%) children with “multi-domain impairment” compared to 10 (15.4%) in the non iGBS group, and 1 (2.9%) child with “moderate to severe impairment” in the exposed group compared to 3 (4.6%) in the non iGBS group. The exposed group had more children with motor impairments compared with the non iGBS group. Studies from South Africa found GBS-affected children to be 13 times more likely to have any abnormal neurological signs when compared with non iGBS group at 6 months (5 [7.4%] in exposed and 1 [0.4%] in non-GBS) [21], and a 3.5-fold (95% CI: 1.23–10.04-fold) increased odds of NDI compared with matched controls at 1 year (11 [24.4%] in exposed and 10 [7.1%] in non iGBS) using abnormal Denver Developmental Screening Test II scores to define NDI [22]. A study on the association of GBS disease on NDI at a median age of 14 years found an increased risk of moderate to severe NDI with risk ratios (RRs) of 1.7 (95% CI: 1.44–2.18) in Denmark and 2.28 (1.64–3.17) in the Netherlands. In Denmark, the proportion of children with moderate to severe NDI at 10 years was 45 (4.6%) in the exposed group and 245 (2.5%) in the non iGBS group (RR: 1.82; 95% CI: 1.33– 2.49). In the Netherlands, the proportion of children who received special educational support at 10 years in the exposed group was 36 (14.3%) and 157 (6.2%) in the non iGBS group [23]. A study in the United States found 2 (16%) children with neonatal GBS to have neurological impairments at 4 years [24]. Children with GBS meningitis showed a larger proportion of NDI: a meta-analysis of GBS meningitis survivors, followed up for more than 18 months reported “any NDI” in 32% (95% CI: 25–38%) of children.

There are several possibilities as to why the rates of moderate to severe NDI in this study were lower than in other studies. The small sample size and the possibility of selection bias (with families of children with less severe impairment more able to attend, especially during the pandemic) may have been factors. In our population, survivors of GBS disease are routinely enrolled in a follow-up program postdischarge, which includes an early stimulation program. Education about developmental milestones may have sensitized the parents to the child’s vulnerability, resulting in active interventions to compensate for the child’s difficulties [25], so the lower rates of impairment may reflect earlier intervention [26]. Systematic reviews have shown that, while early intervention programs may not avert moderate to severe impairment, they have positive effects on cognitive development, with little influence on motor development [27]. Another stipulation is the influence of genetic polymorphisms, such as interleukin-6 (rs1800795), which is associated with the development of cerebral palsy, which may account for racial differences in outcomes of infants seen in other studies [28, 29]. Additionally, there may be measurement error due to varying developmental assessment tools used. The Cognitive, Language, and Motor composites of the BSID-III, for example, have been shown to overestimate development, resulting in lower detection rates of NDI, when compared with the Denver Developmental Screening Test (DDST-II) [30, 31].

In this study, most children with abnormal NDI had language impairments: 64% in “any impairment,” 75% in “moderate to severe impairment,” and 100% in “multi-domain impairment.” This may be explained in 2 ways: the lack of culturally appropriate assessment tools or as inherent cultural differences in language acquisition. The mechanism of language development is proposed to be cultural rather than universal, implying its sensitivity to social factors and cultural context [32].

The non iGBS group in this study has unexpected high percentages of NDI, also reported by (Harden et al, unpublished data) in this series. This study was conducted during the COVID-19 pandemic. The lockdown in India caused a disruption in schooling, with preschoolers not yet enrolled, and most children not having access to any schooling. Since the exposed children were identified and assessed first, and the non iGBS controls recruited later, the effect of the lockdown may have been more pronounced in the non iGBS group. Disasters, including pandemics or disease outbreaks, have been shown to cause short-term and lasting effects on psychological functioning, behavior, and the developmental trajectory of children [33].

The “recovery continuum model” postulates that a child’s recovery after early brain insult falls along a continuum that depends not just on injury-related factors such as nature, severity, and timing of insult but also on constitutional factors such as genetic make-up, gender and cognitive capacity, and environmental factors such as social status, access to rehabilitation, and intervention [34]. A limitation of this study is that, due to the small sample size, confounding factors could not be studied in detail; this can be addressed in future studies in this population.

Strengths of this study include using standardized multicountry approaches and NDI tools with trained assessors, as well as inclusion of a matched non iGBS group. This study was based in a referral center and may therefore not be generalizable to other populations.

### Conclusions

Children with iGBS seem at higher risk of developing motor impairments compared with a non iGBS group. Larger studies are needed in LMICs to estimate incidences of NDI in survivors of GBS and to study environmental adversities that adversely influence child development.

## Supplementary Data

Supplementary materials are available at *Clinical Infectious Diseases* online. Consisting of data provided by the authors to benefit the reader, the posted materials are not copyedited and are the sole responsibility of the authors, so questions or comments should be addressed to the corresponding author.

ciab792_suppl_Supplementary_Materials

## Notes

***Authors’ contributions.*** The GBS study was conceptualized by J. E. L. All site teams contributed to the design of the study protocol and undertook data collection. S. S. together with H. B. J. and A. A. developed the detailed research questions and overall analysis plan for this paper. Analysis was undertaken by G. R. S. S. provided statistical oversight. The manuscript was drafted by H. B. J. and S. S. All authors reviewed and helped to revise the manuscript. All authors reviewed and approved the final version.

***Acknowledgments.*** We thank all the children and their parents who took part in the study. We recognize the contributions of Mr Ramesh Krishnan in managing data, Mr Selvakumar G for translation of consent forms, Ms SujaAngelin and Ms Archana for patient recruitment and data collection, and Ms Poongodi K for her support in data entry. We express appreciation to the GBS LMIC collaborative group for input on the study design as part of a multicountry collaboration.

***Ethics and consent to participate.*** Written informed consent was obtained from parents or guardians and child assent obtained from children over 6 years of age. The overarching protocol for this multicountry observational study was granted ethical approval by the London School of Hygiene and Tropical Medicine (approval number 16246). Institutional review boards at the India site granted ethics approval (India approval numbers: 11723 [Christian Medical College Vellore], 2019–7034 [Indian Council of Medical Research]), as well as the institutional review board of the World Health Organization (approval number ERC.0003169).

***Availability of data and material.*** Data-sharing and transfer agreements were jointly developed and signed by all collaborating partners. The datasets generated and/or analyzed during the current study are not publicly available but are available from the corresponding author on reasonable request.

***Disclaimer.*** The funders had no role in study design, data collection and analysis, decision to publish, or preparation of the manuscript.

***Financial support.*** This work was supported by a grant (INV-009018) from the Bill & Melinda Gates Foundation to the London School of Hygiene and Tropical Medicine (Principal Investigator, J. E. L.). S. S. and G. R. report subawards from London School of Hygiene and Tropical Medicine to Christian Medical College, Vellore. H. B. J. reports that subawards from London School of Hygiene and Tropical Medicine to India were received.

***Supplement sponsorship.*** This supplement is sponsored by Bill & Melinda Gates Foundation.

***Potential conflicts of interest.*** The authors: No reported conflicts of interest. All authors have submitted the ICMJE Form for Disclosure of Potential Conflicts of Interest. Conflicts that the editors consider relevant to the content of the manuscript have been disclosed.

***GBS LMIC Collaborative Group for Long-term Outcomes.*** Shabir A. Madhi (Medical Research Council: Vaccines and Infectious Diseases Analytical Unit, Faculty of Health Sciences, University of the Witwatersrand, Johannesburg, South Africa; Department of Science and Technology/National Research Foundation: Vaccine Preventable Diseases, Faculty of Health Sciences, University of the Witwatersrand, Johannesburg, South Africa), ZiyaadDangor (Medical Research Council: Vaccines and Infectious Diseases Analytical Unit, Faculty of Health Sciences, University of the Witwatersrand, Johannesburg, South Africa), Shannon Leahy (Department of Pediatrics and Child Health, Faculty of Health Sciences, University of the Witwatersrand, Johannesburg, South Africa), Lois Harden (Brain Function Research Group, School of Physiology, Faculty of Health Sciences, University of the Witwatersrand, Johannesburg, South Africa), Azra Ghoor (Department of Pediatrics and Child Health, Faculty of Health Sciences, University of the Witwatersrand, Johannesburg, South Africa), Sibongile Mbatha (Department of Pediatrics and Child Health, Faculty of Health Sciences, University of the Witwatersrand, Johannesburg, South Africa), Sarah Lowick (Department of Pediatrics and Child Health, Faculty of Health Sciences, University of the Witwatersrand, Johannesburg, South Africa), Tamara Jaye (Department of Pediatrics and Child Health, Faculty of Health Sciences, University of the Witwatersrand, Johannesburg, South Africa), Sanjay G. Lala (Department of Pediatrics and Child Health, Faculty of Health Sciences, University of the Witwatersrand, Johannesburg, South Africa), Pamela Sithole (Medical Research Council: Vaccines and Infectious Diseases Analytical Unit, Faculty of Health Sciences, University of the Witwatersrand, Johannesburg, South Africa), Jacqueline Msayi (Medical Research Council: Vaccines and Infectious Diseases Analytical Unit, Faculty of Health Sciences, University of the Witwatersrand, Johannesburg, South Africa), Ntombifuthi Kumalo (Medical Research Council: Vaccines and Infectious Diseases Analytical Unit, Faculty of Health Sciences, University of the Witwatersrand, Johannesburg, South Africa), Tshepiso Nompumelelo Msibi (Medical Research Council: Vaccines and Infectious Diseases Analytical Unit, Faculty of Health Sciences, University of the Witwatersrand, Johannesburg, South Africa), Sridhar Santhanam (Department of Neonatology, Christian Medical College, Vellore, India), Hima B. John (Department of Neonatology, Christian Medical College, Vellore, India), Asha Arumugam (Department of Neonatology, Christian Medical College, Vellore, India), Nandhini Murugesan (Department of Neonatology, Christian Medical College, Vellore, India), Nandhini Rajendraprasad (Department of Neonatology, Christian Medical College, Vellore, India), Mohana Priya (Department of Neonatology, Christian Medical College, Vellore, India), Amina Abubakar (Neuroscience Research Group, Department of Clinical Sciences, KEMRI-Wellcome Trust, Kilifi, Kenya; Institute for Human Development, Aga Khan University, Nairobi, Kenya), Carophine Nasambu (Neuroscience Research Group, Department of Clinical Sciences, KEMRI-Wellcome Trust, Kilifi, Kenya), Adam Mabrouk Adan (KEMRI/Wellcome Trust Research Program, Kilifi, Kenya), Patrick Vidzo Katana (KEMRI/Wellcome Trust Research Program, Kilifi, Kenya), Eva Mwangome (KEMRI/Wellcome Trust Research Program, Kilifi, Kenya), Charles R. Newton (Department of Psychiatry, Medical Sciences Division, University of Oxford, Oxford, UK; KEMRI/Wellcome Trust Research Program), Quique Bassat (Centro de Investigaçãoem Saúde de Manhiça, Maputo, Mozambique; ISGlobal, Hospital Clínic, Universitat de Barcelona, Barcelona, Spain; ICREA, Pg. LluísCompanys 23, Barcelona, Spain; Pediatrics Department, Hospital Sant Joan de Déu [University of Barcelona], Barcelona, Spain; Consorcio de Investigación Biomédicaen Red de Epidemiología y Salud Pública [CIBERESP], Madrid, Spain), Azucena Bardají (Centro de Investigaçãoem Saúde de Manhiça, Maputo, Mozambique; ISGlobal, Hospital Clínic, Universitat de Barcelona, Barcelona, Spain), Justina Bramugy (Centro de Investigaçãoem Saúde de Manhiça, Maputo, Mozambique), Humberto Mucasse (Centro de Investigaçãoem Saúde de Manhiça, Maputo, Mozambique), Celine Aerts (ISGlobal, Hospital Clínic, Universitat de Barcelona, Barcelona, Spain), Sergio Massora (Centro de Investigaçãoem Saúde de Manhiça, Maputo, Mozambique), Romina Libster (Fundación INFANT, Buenos Aires, Argentina), Clara Sánchez Yanotti (Fundación INFANT, Buenos Aires, Argentina), Valeria Medina (Instituto de Maternidad y Ginecología Nuestra Señora de las Mercedes, Argentina), Andrea Rojas (Instituto de Maternidad y Ginecología Nuestra Señora de las Mercedes, Argentina), Daniel Amado (Instituto de Maternidad y Ginecología Nuestra Señora de las Mercedes, Argentina), Conrado J. Llapur (Instituto de Maternidad y Ginecología Nuestra Señora de las Mercedes, Argentina), A. K. M. Tanvir Hossain (Maternal and Child Health Division, International Centre for Diarrhoeal Disease Research, Bangladesh [icddr,b], Dhaka, Bangladesh), QaziSadeq-ur Rahman (Maternal and Child Health Division, icddr,b, Dhaka, Bangladesh).

## References

[1] Seale AC , Blencowe H, Manu AA, et al.; pSBI Investigator Group. Estimates of possible severe bacterial infection in neonates in sub-Saharan Africa, South Asia, and Latin America for 2012: a systematic review and meta-analysis. Lancet Infect Dis 2014; 14:731–41. 24974250 10.1016/S1473-3099(14)70804-7 PMC4123782

[2] Iroh Tam PY , Delair SF, Obaro SK. Neonatal group B streptococcus disease in developing countries: are we ready to deploy a vaccine? Expert Rev Vaccines 2015; 14:1401–3. 26289974 10.1586/14760584.2015.1077121

[3] Madrid L , Seale AC, Kohli-Lynch M, et al.; Infant GBS Disease Investigator Group. Infant group B streptococcal disease incidence and serotypes worldwide: systematic review and meta-analyses. Clin Infect Dis 2017; 65:160–72. 10.1093/cid/cix656 PMC5850457 29117326

[4] Mwaniki MK , Atieno M, Lawn JE, Newton CR. Long-term neurodevelopmental outcomes after intrauterine and neonatal insults: a systematic review. Lancet 2012; 379:445–52. 22244654 10.1016/S0140-6736(11)61577-8 PMC3273721

[5] Hernández MI , Sandoval CC, Tapia JL, et al. Stroke patterns in neonatal group B streptococcal meningitis. Pediatr Neurol 2011; 44:282–8. 21397170 10.1016/j.pediatrneurol.2010.11.002

[6] Seale AC , Blencowe H, Zaidi A, et al; Neonatal Infections Estimation Team. Neonatal severe bacterial infection impairment estimates in South Asia, sub-Saharan Africa, and Latin America for 2010. Pediatr Res 2013; 74(Suppl 1):73–85. 24366464 10.1038/pr.2013.207 PMC3873707

[7] Kohli-Lynch M , Russell NJ, Seale AC, et al. Neurodevelopmental impairment in children after group B streptococcal disease worldwide: systematic review and meta-analyses. Clin Infect Dis 2017; 65:190–9. 10.1093/cid/cix663 PMC5848372 29117331

[8] Santhanam S , Beck MM, Veeraraghavan B, et al. Group B Streptococcus (GBS) colonization in mother-newborn dyads in India—results from a multicentre study. In: Proceedings of the 1st International Symposium of Streptococcus Agalactiae Disease (ISSAD), 21–23 February 2018, Cape Town, South Africa.

[9] Sridhar S , Grace R, Nithya PJ, et al. Group B streptococcal infection in a tertiary hospital in India—1998-2010. Pediatr Infect Dis J 2014; 33:1091–2. 24776515 10.1097/INF.0000000000000377

[10] Paul P , Procter SR, Dangor Z, et al. Quantifying long-term health and economic outcomes for survivors of group B Streptococcus invasive disease in infancy: protocol of a multi-country study in Argentina, India, Kenya, Mozambique and South Africa. Gates Open Res 2020; 4:138. 34368637 10.12688/gatesopenres.13185.1 PMC8313848

[11] Upadhyay RP , Taneja S, Ranjitkar S, et al. Factors determining cognitive, motor and language scores in low birth weight infants from North India. PLoS One 2021; 16:e0251387. 33979366 10.1371/journal.pone.0251387 PMC8115769

[12] Andrew A , Attanasio O, Augsburg B, et al. Effects of a scalable home-visiting intervention on child development in slums of urban India: evidence from a randomised controlled trial. J Child Psychol Psychiatry 2020; 61:644–52. 31797385 10.1111/jcpp.13171 PMC7242140

[13] Bhopal S , Roy R, Verma D, et al. Impact of adversity on early childhood growth & development in rural India: findings from the early life stress sub-study of the SPRING cluster randomised controlled trial (SPRING-ELS). PLoS One 2019; 14:e0209122. 30625145 10.1371/journal.pone.0209122 PMC6326522

[14] Wechsler D. Wechsler preschool and primary scale of intelligence—fourth edition. San Antonio, TX: Psychol Corp, 2012.

[15] Ruan-Iu L , Pendergast LL, Rasheed M, et al. Assessing early childhood fluid reasoning in low-and middle-income nations: validity of the Wechsler Preschool and Primary Scale of Intelligence across seven MAL-ED sites. J Psychoeduc Assess 2020; 38:256–62.

[16] Wechsler D IV . WISC-IV India. Wechsler Intelligence Scale for Children–fourth (India edition). New Delhi, India: Pearson, 2016.

[17] Brown T . Structural validity of the Bruininks-Oseretsky test of motor proficiency—second edition brief form (BOT-2-BF). Res Dev Disabil 2019; 85:92–103. 30502549 10.1016/j.ridd.2018.11.010

[18] Achenbach TM , Rescorla LA. Manual for the ASEBA preschool forms and profiles. Vol 30. Burlington, VT: Universityof Vermont, Research Center for Children, Youth, 2000.

[19] Achenbach TM , Rescorla L. Manual for the ASEBA school-age forms & profiles: an integrated system of multi-informant assessment. Burlington, VT: ASEBA, 2001.

[20] Saleem SM . Modified Kuppuswamy socioeconomic scale updated for the year 2020. Indian J Forensic Community Med 2020; 7:1–3.

[21] Dangor Z , Lala SG, Cutland CL, et al. Burden of invasive group B Streptococcus disease and early neurological sequelae in South African infants. PLoS One 2015; 10:e0123014. 25849416 10.1371/journal.pone.0123014 PMC4388823

[22] Nakwa FL , Lala SG, Madhi SA, Dangor Z. Neurodevelopmental impairment at 1 year of age in infants with previous invasive group B Streptococcal sepsis and meningitis. Pediatr Infect Dis J 2020; 39:794–8. 32804460 10.1097/INF.0000000000002695

[23] Horváth-Puhó E , van Kassel MN, Gonçalves BP, et al. Mortality, neurodevelopmental impairments, and economic outcomes after invasive group B streptococcal disease in early infancy in Denmark and the Netherlands: a national matched cohort study. Lancet Child Adolesc Health 2021; 5:398–407. 33894156 10.1016/S2352-4642(21)00022-5 PMC8131199

[24] Horn KA , Zimmerman RA, Knostman JD, Meyer WT. Neurological sequelae of group B streptococcal neonatal infection. Pediatrics 1974; 53:501–4. 4132678

[25] Gueron-Sela N , Atzaba-Poria N, Meiri G, Marks K. The caregiving environment and developmental outcomes of preterm infants: diathesis stress or differential susceptibility effects? Child Dev 2015; 86:1014–30. 25875941 10.1111/cdev.12359

[26] Oommen SP , Santhanam S, John H, et al. Neurodevelopmental outcomes of very low birth weight infants at 18-24 months, corrected gestational age in a tertiary health centre: a prospective cohort study. J Trop Pediatr 2019; 65:552–60. 30793756 10.1093/tropej/fmz006

[27] Spittle AJ , Morgan C, Olsen JE, Novak I, Cheong JLY. Early diagnosis and treatment of cerebral palsy in children with a history of preterm birth. Clin Perinatol 2018; 45:409–20. 10.1016/j.clp.2018.05.011 30144846

[28] Nelson KB , Dambrosia JM, Iovannisci DM, Cheng S, Grether JK, Lammer E. Genetic polymorphisms and cerebral palsy in very preterm infants. Pediatr Res 2005; 57:494–9. 15718364 10.1203/01.PDR.0000156477.00386.E7

[29] Wu D , Zou YF, Xu XY, et al. The association of genetic polymorphisms with cerebral palsy: a meta-analysis. Dev Med Child Neurol 2011; 53:217–25. 21291465 10.1111/j.1469-8749.2010.03884.x

[30] Anderson PJ , Burnett A. Assessing developmental delay in early childhood—concerns with the Bayley-III scales. Clin Neuropsychol 2017; 31:371–81. 27687612 10.1080/13854046.2016.1216518

[31] Jeong SU , Kim GC, Jeong HJ, et al. The Validity of the Bayley-III and DDST-II in preterm infants with neurodevelopmental impairment: a pilot study. Ann Rehabil Med 2017; 41:851–7. 29201825 10.5535/arm.2017.41.5.851 PMC5698673

[32] Fitneva SA , Matsui T. The emergence and development of language across cultures. In: Jensen LA, ed. The Oxford handbook of human development and culture: An interdisciplinary perspective. Oxford University Press, 2015:111–26.

[33] Schonfeld DJ , Demaria T; Disaster Preparedness Advisory Council and Committee on Psychosocial Aspects of Child and Family Health. Providing psychosocial support to children and families in the aftermath of disasters and crises. Pediatrics 2015; 136:e1120–30. 26371193 10.1542/peds.2015-2861

[34] Anderson V , Spencer-Smith M, Wood A. Do children really recover better? Neurobehavioural plasticity after early brain insult. Brain 2011; 134:2197–221. 21784775 10.1093/brain/awr103

