# Supplementary material for: South Indian Children’s Neurodevelopmental Outcomes After Group B Streptococcus Invasive Disease: A Matched-Cohort Study
Source: Clin Infect Dis. 2021 Nov 3;74(Suppl 1):S24–34. doi: 10.1093/cid/ciab792 (PMC8775645; doi:10.1093/cid/ciab792)
Supplement: ciab792_suppl_Supplementary_Materials [file ciab792_suppl_supplementary_materials.docx]

**Supplement Title**: Every Country, Every Woman, Every Child; Group B Streptococcal Disease Worldwide

**Title:** South Indian children’s neurodevelopmental outcomes after Group B Streptococcus invasive disease and associations with socioeconomic status: A case cohort study

**Short title:** South Indian children outcomes after iGBS

**Authors:** Hima B John^1^, Asha Arumugam^1^, Mohana Priya^1^, Nandhini Murugesan^1^, Nandhini Rajendraprasad^1^, Grace Rebekah^2^, Proma Paul^3,4^, Jaya Chandna^3,4^, Joy E Lawn^3,4^, Santhanam Sridhar^1*^

*Corresponding author

**Affiliations:**

1. Department of Neonatology, Christian Medical College, Vellore
2. Department of Biostatistics, Christian Medical College, Vellore
3. Maternal, Adolescent, Reproductive & Child Health (MARCH) Centre, London School of Hygiene & Tropical Medicine, London, UK
4. Department of Infectious Disease Epidemiology, London School of Hygiene & Tropical Medicine, London, UK

Table of Contents

[**Supplemental Table 1:** STROBE Statement—Checklist of items that should be included in reports of cohort studies 3](#_Toc81472396)

[**Supplementary Table 2:** Definitions of variables used 5](#_Toc81472397)

[REFERENCES 6](#_Toc81472398)

# **Supplemental Table 1:** STROBE Statement—Checklist of items that should be included in reports of cohort studies

|  | Item No | Recommendation | Page No |
| --- | --- | --- | --- |
| **Title and abstract** | 1 | (*a*) Indicate the study’s design with a commonly used term in the title or the abstract | 1 |
|  |  | (*b*) Provide in the abstract an informative and balanced summary of what was done and what was found | 2 |
| Introduction | | | |
| Background/rationale | 2 | Explain the scientific background and rationale for the investigation being reported | 4 |
| Objectives | 3 | State specific objectives, including any prespecified hypotheses | 4-5 |
| Methods | | | |
| Study design | 4 | Present key elements of study design early in the paper | 5 |
| Setting | 5 | Describe the setting, locations, and relevant dates, including periods of recruitment, exposure, follow-up, and data collection | 5 |
| Participants | 6 | (*a*) Give the eligibility criteria, and the sources and methods of selection of participants. Describe methods of follow-up | 5 |
|  |  | (*b*)For matched studies, give matching criteria and number of exposed and unexposed | 5 |
| Variables | 7 | Clearly define all outcomes, exposures, predictors, potential confounders, and effect modifiers. Give diagnostic criteria, if applicable | 6-7 |
| Data sources/ measurement | 8* | For each variable of interest, give sources of data and details of methods of assessment (measurement). Describe comparability of assessment methods if there is more than one group | 6-7 |
| Bias | 9 | Describe any efforts to address potential sources of bias | 6 |
| Study size | 10 | Explain how the study size was arrived at | 5 |
| Quantitative variables | 11 | Explain how quantitative variables were handled in the analyses. If applicable, describe which groupings were chosen and why | 7-8 |
| Statistical methods | 12 | (*a*) Describe all statistical methods, including those used to control for confounding | 7-8 |
|  |  | (*b*) Describe any methods used to examine subgroups and interactions |  |
|  |  | (*c*) Explain how missing data were addressed |  |
|  |  | (*d*) If applicable, explain how loss to follow-up was addressed |  |
|  |  | (*e*) Describe any sensitivity analyses |  |
| Results | | |  |
| Participants | 13* | (a) Report numbers of individuals at each stage of study—eg numbers potentially eligible, examined for eligibility, confirmed eligible, included in the study, completing follow-up, and analysed | Figure 2, Page 8 |
|  |  | (b) Give reasons for non-participation at each stage |  |
|  |  | (c) Consider use of a flow diagram |  |
| Descriptive data | 14* | (a) Give characteristics of study participants (eg demographic, clinical, social) and information on exposures and potential confounders | 8 |
|  |  | (b) Indicate number of participants with missing data for each variable of interest |  |
|  |  | (c) Summarise follow-up time (eg, average and total amount) |  |
| Outcome data | 15* | Report numbers of outcome events or summary measures over time | 8-9 |

| Main results | 16 | (*a*) Give unadjusted estimates and, if applicable, confounder-adjusted estimates and their precision (eg, 95% confidence interval). Make clear which confounders were adjusted for and why they were included | NA |
| --- | --- | --- | --- |
|  |  | (*b*) Report category boundaries when continuous variables were categorized |  |
|  |  | (*c*) If relevant, consider translating estimates of relative risk into absolute risk for a meaningful time period |  |
| Other analyses | 17 | Report other analyses done—eg analyses of subgroups and interactions, and sensitivity analyses | NA |
| Discussion | | | |
| Key results | 18 | Summarise key results with reference to study objectives | 10 |
| Limitations | 19 | Discuss limitations of the study, taking into account sources of potential bias or imprecision. Discuss both direction and magnitude of any potential bias | 13 |
| Interpretation | 20 | Give a cautious overall interpretation of results considering objectives, limitations, multiplicity of analyses, results from similar studies, and other relevant evidence | 10- 13 |
| Generalisability | 21 | Discuss the generalisability (external validity) of the study results | 13 |
| Other information | | | |
| Funding | 22 | Give the source of funding and the role of the funders for the present study and, if applicable, for the original study on which the present article is based | 15 |

*Give information separately for exposed and unexposed groups.

# **Supplementary Table 2:**Definitions of variables used

| Variables | Definitions |
| --- | --- |
| iGBS disease | Exposure to GBS disease was defined as GBS isolated in blood culture between 0-89 days of the infant’s life. |
| Severity coding for neurodevelopmental impairment | |
| Mild | 1-2 SD below standardised mean |
| Moderate | 2-3 SD below standardised mean |
| Severe | ≥ 3 SD below standardised mean |
| Any impairment | Any degree of impairment in any domain (vision, hearing, cognition, language, motor or behaviour) |
| Multi- domain impairment | Any degree of impairment in more than one domain(vision, hearing, cognition, language, motor or behaviour) |
| Moderate to severe impairment | Moderate or severe impairment in any domain (vision, hearing, cognitionor motor domains) |
| Combined analysis of cohort | |
| Cognition domain | Cognition subscale of BSID and Fullscale IQs of WISC or WPPSI |
| Language domain | Language subscale of BSID and verbal comprehension subscale of WISC or WPPSI |
| Motor domain | Motor subscale of BSID and the interpretation of the BOT-2 |
| Behavioural domain | CBCL and Socio- emotional scale of BSID (for children below 18 months) |

# REFERENCES

1. Seale AC, Blencowe H, Manu AA, et al. Estimates of possible severe bacterial infection in neonates in sub-Saharan Africa, south Asia, and Latin America for 2012: a systematic review and meta-analysis. *Lancet Infect Dis*. 2014;14(8):731-741.

2. Iroh Tam P-Y, Delair SF, Obaro SK. Neonatal group B streptococcus disease in developing countries: are we ready to deploy a vaccine? *Expert Rev Vaccines*. 2015;14(11):1401-1403.

3. Madrid L, Seale AC, Kohli-Lynch M, et al. Infant group B streptococcal disease incidence and serotypes worldwide: systematic review and meta-analyses. *Clin Infect Dis*. 2017;65(suppl_2):S160-S172.

4. Mwaniki MK, Atieno M, Lawn JE, Newton CR. Long-term neurodevelopmental outcomes after intrauterine and neonatal insults: a systematic review. *The Lancet*. 2012;379(9814):445-452.

5. Hernández MI, Sandoval CC, Tapia JL, et al. Stroke patterns in neonatal group B streptococcal meningitis. *Pediatr Neurol*. 2011;44(4):282-288.

6. Seale AC, Blencowe H, Zaidi A, et al. Neonatal severe bacterial infection impairment estimates in South Asia, sub-Saharan Africa, and Latin America for 2010. *Pediatr Res*. 2013;74(1):73-85.

7. Kohli-Lynch M, Russell NJ, Seale AC, et al. Neurodevelopmental impairment in children after group B streptococcal disease worldwide: systematic review and meta-analyses. *Clin Infect Dis*. 2017;65(suppl_2):S190-S199.

8. Santhanam S, Beck M et al.Group B Streptococcus (GBS) colonization in mother-newborn dyads in India– Results from a multicentre study. In: Proceedings of the 1st International Symposium of Streptococcus agalactiae disease (ISSAD), Feb 21-23, 2018, Cape Town, South Africa

9. Sridhar S, Grace R, Nithya PJ, et al. Group B streptococcal infection in a tertiary hospital in India—1998–2010. *Pediatr Infect Dis J*. 2014;33(10):1091-1092.

10. Paul P, Procter SR, Dangor Z, et al. Quantifying long-term health and economic outcomes for survivors of group B Streptococcus invasive disease in infancy: protocol of a multi-country study in Argentina, India, Kenya, Mozambique and South Africa. *Gates Open Res*. 2020;4(138):138.

11. Upadhyay RP, Taneja S, Ranjitkar S, et al. Factors determining cognitive, motor and language scores in low birth weight infants from North India. *Plos One*. 2021;16(5):e0251387.

12. Andrew A, Attanasio O, Augsburg B, et al. Effects of a scalable home-visiting intervention on child development in slums of urban India: evidence from a randomised controlled trial. *J Child Psychol Psychiatry*. 2020;61(6):644-652.

13. Bhopal S, Roy R, Verma D, et al. Impact of adversity on early childhood growth & development in rural India: Findings from the early life stress sub-study of the SPRING cluster randomised controlled trial (SPRING-ELS). *PLoS One*. 2019;14(1):e0209122.

14. Wechsler D. Wechsler preschool and primary scale of intelligence—fourth edition. *Psychol Corp San Antonio TX*. Published online 2012.

15. Ruan-Iu L, Pendergast LL, Rasheed M, et al. Assessing early childhood fluid reasoning in low-and middle-income nations: validity of the Wechsler Preschool and Primary Scale of Intelligence across seven MAL-ED sites. *J Psychoeduc Assess*. 2020;38(2):256-262.

16. Wechsler IV D. *WISC-IV India. Wechsler Intelligence Scale for Children-Fourth (India Edition)*. New Delhi: Pearson; 2016.

17. Brown T. Structural validity of the Bruininks-Oseretsky test of motor proficiency–second edition brief form (BOT-2-BF). *Res Dev Disabil*. 2019;85:92-103.

18. Achenbach TM, Rescorla LA. *Manual for the ASEBA Preschool Forms and Profiles*. Vol 30. Burlington, VT: University of Vermont, Research center for children, youth …; 2000.

19. Achenbach TM, Rescorla L. *Manual for the ASEBA School-Age Forms & Profiles: An Integrated System of Multi-Informant Assessment*. Aseba Burlington, VT:; 2001.

20. Saleem SM. Modified Kuppuswamy socioeconomic scale updated for the year 2020. *Indian J Forensic Community Med*. 2020;7(1):1-3.

21. Dangor Z, Lala SG, Cutland CL, et al. Burden of invasive group B Streptococcus disease and early neurological sequelae in South African infants. *PloS One*. 2015;10(4):e0123014.

22. Nakwa FL, Lala SG, Madhi SA, Dangor Z. Neurodevelopmental Impairment at 1 Year of Age in Infants With Previous Invasive Group B Streptococcal Sepsis and Meningitis. *Pediatr Infect Dis J*. 2020;39(9):794-798.

23. Horváth-Puhó E, van Kassel MN, Gonçalves BP, et al. Mortality, neurodevelopmental impairments, and economic outcomes after invasive group B streptococcal disease in early infancy in Denmark and the Netherlands: a national matched cohort study. *Lancet Child Adolesc Health*. 2021;5(6):398-407.

24. Horn KA, Zimmerman RA, Knostman JD, Meyer WT. Neurological sequelae of group B streptococcal neonatal infection. *Pediatrics*. 1974;53(4):501-504.

25. Gueron-Sela N, Atzaba-Poria N, Meiri G, Marks K. The caregiving environment and developmental outcomes of preterm infants: Diathesis stress or differential susceptibility effects? *Child Dev*. 2015;86(4):1014-1030.

26. Oommen SP, Santhanam S, John H, et al. Neurodevelopmental Outcomes of Very Low Birth Weight Infants at 18–24 Months, Corrected Gestational Age in a Tertiary Health Centre: A Prospective Cohort Study. *J Trop Pediatr*. Published online 2019.

27. Spittle AJ, Morgan C. Early intervention for children with cerebral palsy. In: *Cerebral Palsy*. Springer; 2018:193-200.

28. Nelson KB, Dambrosia JM, Iovannisci DM, Cheng S, Grether JK, Lammer E. Genetic polymorphisms and cerebral palsy in very preterm infants. *Pediatr Res*. 2005;57(4):494.

29. Wu D, ZOU Y-F, XU X-Y, et al. The association of genetic polymorphisms with cerebral palsy: a meta-analysis. *Dev Med Child Neurol*. 2011;53(3):217-225.

30. Anderson PJ, Burnett A. Assessing developmental delay in early childhood—concerns with the Bayley-III scales. *Clin Neuropsychol*. 2017;31(2):371-381.

31. Jeong SU, Kim GC, Jeong HJ, et al. The Validity of the Bayley-III and DDST-II in preterm infants with neurodevelopmental impairment: a pilot study. *Ann Rehabil Med*. 2017;41(5):851.

32. Fitneva SA, Matsui T. The emergence and development of language across cultures. Published online 2015.

33. Schonfeld DJ, Demaria T. Providing psychosocial support to children and families in the aftermath of disasters and crises. *Pediatrics*. 2015;136(4):e1120-e1130.

34. Anderson V, Spencer-Smith M, Wood A. Do children really recover better? Neurobehavioural plasticity after early brain insult. *Brain*. 2011;134(8):2197-2221.
